# English Isolation Hospitals

**Published:** 1892-01-02

**Authors:** 


### English Isolation Hospitals.

A paper read at the International Congress of Hygiene by Dr. Thorne Thorne on the above subject is of considerable importance. During the last twenty years great interest has been taken in the question of public health, and especially in the prevention of diseases. In 1871 the Local Government Board was established, and, among the many duties which it had to perform was that of looking after the public health. Since that year many Acts of Parliament have been passed to prevent the spread of the disease, *e.g.*, the Public Health Act, 1873, the Sale of Food and Drugs Act, the Housing of the Working Classes Acts, the Notification of Diseases Acts, &c.

Few persons have any idea of the immense work which the Local Government Board has accomplished, and of the very great reforms in the matter of public health which have been carried on under its auspices. Some notion of what has been done may be gained from the large sums of money, of which it has sanctioned the borrowing, for sanitary and health purposes by various local authorities. From the time of its constitution to the end of 1890, it had sanctioned the borrowing of £46,762,583 for sanitary improvements throughout England and Wales, and £43,473,812 for sanitary purposes other than improvement schemes. In addition to this, the borrowing of £50,918,304 by sanitary authorities has been allowed by Act of Parliament, so that during the nineteen years ended December 31, 1891, the spending of £140,000,000 has been sanctioned for public health purposes. The greater part of this was for sewage disposals, water supply, hospitals for infectious diseases, etc. There can be no doubt but that to these great sanitary improvements the decrease in the death-rate is to be attributed. In London, the death-rate in 1871 was 24·7 per 1,000. In 1890 it was reduced to 20·3.

The supreme usefulness of Isolation Hospitals in preventing the spread of zymotic diseases admits of no doubt. Dr. Thorne Thorne tells us that during the last eleven years quite a hundred new Isolation Hospitals have been built by different local authorities, making about four hundred in all for England and Wales. He thinks, too, that the adoption of the permissive clauses of the Infectious Diseases (Notification) Act, 1889, is likely to lead to a substantial increase in their number. Dr. Thorne Thorne says, "So far there is no evidence that such aggregation of the infectious sick suffering from any of these diseases, except small-pox, as is likely to be carried out by any ordinary sanitary authority, leads to diffusion of infection to the surrounding community, provided the hospitals be properly constructed and administered, and subject to a zone of some forty feet being provided between all buildings intended to receive infected persons or things, and the boundary wall, or closed fence around the site. Experience has shown that it is otherwise with small-pox. . . ." In Appendix C. of the Local Government Board report for 1885, is a memorandum by Dr. Bridges, the Medical Inspector, in which an opposite view is taken. There are in fact two sides of the question, Dr. Bridges there considers the question of the spread of small-pox by the atmosphere, as not proved, the suggestion being that any increase of the disease in the neighbourhood of the hospital is to be accounted for by inefficient seclusion, "human communication," rather than by atmospheric influence.

The infectious diseases which specially call for isolation are in order of their importance, scarlet fever, typhus, small-pox, diphtheria, typhoid fever, and cholera; and to these may be added, as being less frequently isolated in hospital, erysipelas, measles, whooping-cough, and puerperal fever. Diphtheria has only recently been admitted into the Metropolitan Asylums Board hospitals. There can be no doubt but that it would be for the benefit of the community if all the infectious diseases were isolated. With measles there is the difficulty that the pre-eruptive stage is highly infectious, and that before isolation can be effected, other persons will have probably caught the infection. Still, the spread of measles, and also of erysipelas, whooping-cough, and puerperal fever would no doubt be greatly lessened by isolation. The mortality from these latter diseases is very large, especially when compared with the first. In London, in 1890, the deaths from the two classes were as follows:—

Scarlet fever, 876; typhus, 11; small-pox, 4; simple cholera, 83; diphtheria, 1,417; typhoid, 618; total, 3,009.

Erysipelas, 250; measles, 3,291; whooping-cough, 3,276; puerperal fever, 237; total, 7,054.

A feeling exists that every child should have measles and whooping-cough; and nothing whatever is done to prevent them, in spite of the large mortality. If the former great small-pox mortality can be lowered to four, why not that of measles and whooping-cough? It must be remembered that this same feeling of doing nothing once existed with regard to small-pox, but it has rightly given way to more efficacious methods.

Dr. Thorne Thorne's paper deals with the principal points to be had in view in providing for the erection of infectious hospitals, and it may be considered as a text-book of the subject. Being Assistant Medical Officer to the Local Government Board, he speaks with authority

